# Transient B-Cell Depletion with Anti-CD20 in Combination with Proinsulin DNA Vaccine or Oral Insulin: Immunologic Effects and Efficacy in NOD Mice

**DOI:** 10.1371/journal.pone.0054712

**Published:** 2013-02-06

**Authors:** Ghanashyam Sarikonda, Sowbarnika Sachithanantham, Yulia Manenkova, Tinalyn Kupfer, Amanda Posgai, Clive Wasserfall, Philip Bernstein, Laura Straub, Philippe P. Pagni, Darius Schneider, Teresa Rodriguez Calvo, Marilyne Coulombe, Kevan Herold, Ronald G. Gill, Mark Atkinson, Gerald Nepom, Mario Ehlers, Teodora Staeva, Hideki Garren, Lawrence Steinman, Andrew C. Chan, Matthias von Herrath

**Affiliations:** 1 La Jolla Institute for Allergy and Immunology, Diabetes Center, La Jolla, California, United States of America; 2 University of Colorado Denver, Aurora, Colorado, United States of America; 3 University of Florida, Gainesville, Florida, United States of America; 4 Immune Tolerance Network, San Francisco, California, United States of America; 5 JDRF International, New York, New York, United States of America; 6 Bayhill Therapeutics, San Mateo, California, United States of America; 7 Stanford University, Stanford, California, United States of America; 8 Genentech, Inc, South San Francisco, California, United States of America; 9 Yale University, New Haven, Connecticut, United States of America; 10 Benaroya Research Institute, Seattle, Washington, United States of America; Children’s Hospital Boston/Harvard Medical School, United States of America

## Abstract

A recent type 1 diabetes (T1D) clinical trial of rituximab (a B cell-depleting anti-CD20 antibody) achieved some therapeutic benefit in preserving C-peptide for a period of approximately nine months in patients with recently diagnosed diabetes. Our previous data in the NOD mouse demonstrated that co-administration of antigen (insulin) with anti-CD3 antibody (a T cell-directed immunomodulator) offers better protection than either entity alone, indicating that novel combination therapies that include a T1D-related autoantigen are possible. To accelerate the identification and development of novel combination therapies that can be advanced into the clinic, we have evaluated the combination of a mouse anti-CD20 antibody with either oral insulin or a proinsulin-expressing DNA vaccine. Anti-CD20 alone, given once or on 4 consecutive days, produced transient B cell depletion but did not prevent or reverse T1D in the NOD mouse. Oral insulin alone (twice weekly for 6 weeks) was also ineffective, while proinsulin DNA (weekly for up to 12 weeks) showed a trend toward modest efficacy. Combination of anti-CD20 with oral insulin was ineffective in reversing diabetes in NOD mice whose glycemia was controlled with SC insulin pellets; these experiments were performed in three independent labs. Combination of anti-CD20 with proinsulin DNA was also ineffective in diabetes reversal, but did show modest efficacy in diabetes prevention (p = 0.04). In the prevention studies, anti-CD20 plus proinsulin resulted in modest increases in Tregs in pancreatic lymph nodes and elevated levels of proinsulin-specific CD4+ T-cells that produced IL-4. Thus, combination therapy with anti-CD20 and either oral insulin or proinsulin does not protect hyperglycemic NOD mice, but the combination with proinsulin offers limited efficacy in T1D prevention, potentially by augmentation of proinsulin-specific IL-4 production.

## Introduction

In type 1 diabetes (T1D) antigen-specific immunotherapy (ASI) is a desirable goal because it offers the prospect of inducing immune tolerance with a good safety profile [1]. To date, however, clinical trials of ASI in the prevention or treatment of T1D have shown little or no efficacy, despite encouraging preclinical data. Success in the clinic may require optimization of dose, frequency, route of administration, and choice of antigen/epitope and adjuvant [2]. In addition, it is possible that in human T1D, ASI alone is not sufficient to induce tolerance but requires combination with an appropriate immune modulator that can enhance regulatory T cell (Treg) function and reduce the load of effector cells. This approach was recently validated in the NOD mouse, in which combination of non-Fc receptor binding anti-CD3 Mab with nasal proinsulin was more effective in reversing diabetes than either agent alone [3]. This has prompted strong interest in combination therapies, particularly those in which the individual components have already shown safety or efficacy in human trials [4]. Based on these considerations we explored the combination of an insulin-based antigen with anti-CD20 Mab in the NOD mouse.

Among ASI options for T1D, antigens based on insulin have received the most attention in the clinic. Both oral and nasal insulin have been evaluated in T1D prevention trials [5], [6], while nasal insulin, DNA encoding proinsulin, proinsulin peptide, and insulin B-chain formulated in adjuvant have been administered in new-onset and established T1D [7]–[10]. Overall, results have been disappointing but there have been signals of efficacy in defined subpopulations as well as encouraging immunologic changes; safety and tolerability have been good, with no signs of disease exacerbation. Insulin is an important auto-antigen in human T1D and a high proportion of auto-reactive, islet-infiltrating CD8 T cells, which selectively destroy insulin producing β-cells [11], are insulin-reactive [12]. Insulin is also the primary antigen leading to targeted islet cell destruction in the NOD mouse [13]. In mouse models, administration of insulin or insulin peptides increases the numbers of antigen-specific Treg cells that can prevent T1D [14]–[16]. DNA vaccination with insulin B-chain prevented diabetes onset in NOD [17] and RIP-NP mice [18] through a mechanism involving IL-4 production [17], [18], and administration of a DNA vaccine encoding proinsulin was effective in both prevention and reversal of diabetes in NOD mice [9].

Among antigen-nonspecific, targeted immunomodulation approaches for T1D, many have been evaluated in the clinic (reviewed in [2]) but thus far only three have shown a sign of efficacy in well-controlled phase 2 trials: FcR-nonbinding anti-CD3 Mab [19], [20], anti-CD20 Mab [21], and CTLA4-Ig [22]. While anti-CD3 treatment appears to exert its effect through the induction of IL-10-producing Tregs [23], the mechanism of action of anti-CD20 is not clear. B-cells participate in most autoimmune diseases through production of autoantibodies [24], but in T1D they likely promote disease by functioning as antigen presenting cells (APCs) that specifically and efficiently capture beta-cell proteins, including insulin [25]–[29]. Studies in NOD mice using anti-CD20 antibody have shown contrasting effects of B-cell depletion on reversal or prevention of T1D [30]–[32]. While one group found induction of regulatory B-cells (Bregs) and Tregs [30], another group did not [32]. In reports that used nontransgenic NOD mice, therapeutic efficacy of B-cell depletion on T1D after onset was not observed [32]. B-cells that infiltrate the pancreas may lose CD20 expression and gain a plasma cell phenotype, which could explain the loss of anti-CD20-mediated protection in mice at later stages of the disease [31]. In transgenic NOD mice, transient anti-CD20 treatment in very young mice (newborn), but not in older mice, prevented autoimmune disease onset later in life [33]. Collectively, the data suggest that antigen-specific B cells facilitate and enhance disease development and progression in T1D.

In this study, we hypothesized that co-administration of a B cell-depleting anti-CD20 Mab and either a proinsulin DNA vaccine or oral insulin will offer synergistic protection before and after T1D onset in NOD mice.

## Materials and Methods

### Mice Maintenance

NOD/LtJ, NOD.SCID (NOD.CB17-*Prkdc^scid^*/J) or C57BL/6 mice were purchased from the Jackson Laboratory (Bar Harbor, ME, USA). NOD/LtJ and C57BL/6 mice were maintained in specific pathogen-free conditions at the three participating institutions while NOD.SCID mice were maintained in a sterile suite at the La Jolla Institute for Allergy and Immunology animal facility.

For animal care and use at LIAI, La Jolla Institute For Allergy And Immunology Animal Care Committee approved the work carried out in this manuscript, under our protocol titled: Immune Mediated Diabetes, Reference: Ap151-Mvh8-510. Animal welfare is governed by the guidelines established through “The Guide” (The Guide For The Care And Use Of Laboratory Animals) and the AVMA panel on euthanasia.

For animal care and use at UC Denver, mice were housed, cared for, and used in experiments in accordance with the eighth edition of the Guide for the Care and Use of Laboratory Animals as described in protocol B-75411(05)1E and as approved by the University of Colorado’s Institutional Animal Care and Use Committee. The IACUC-approved pain management administered following insulin pellet implantation was the opioid analgesic buprenorphine. Buprenorphine (0.2 mg/kg SC) was given just prior to surgery and continued every 6–12 hours for the next 18–24 hours based on pain assessment. The IACUC-approved pain management administered following retro-orbital blood collection was proparacaine ophthalmic solution. Isoflurane anesthesia was administered prior to both procedures. The IACUC-approved euthanasia method during this study was CO_2_ asphyxiation followed by cervical dislocation.

For animal care and use at University of Florida, mice were housed, cared for, and used in experiments in accordance with the Guide for the Care and Use of Laboratory Animals as approved by the University of Florida’s Institutional Animal Care and Use Committee. The IACUC-approved anesthesia administered prior to and during insulin pellet implantation was isoflurane inhalation (5% induction and 1–2% maintenance checked by toe pinch reflex). Tail vein blood collections were performed rather than retro-orbital blood collection; thus, a pain management solution was not needed for blood collections. The IACUC-approved euthanasia method during this study was isoflurane inhalation. The toe pinch reflex was used to confirm that the animal is completely unconscious. Cardiac exsanguination was performed while under deep anesthesia followed by cervical dislocation. At this point, thoracotomy with full necropsy was performed.

### Blood Glucose Monitoring

Blood glucose values (BGV) were monitored biweekly with OneTouch Ultra blood glucose monitoring system (LifeScan Inc., Milpitas, CA, USA). For studies involving the combination of anti-CD20 and oral insulin, NOD mice were considered to be diabetic when two consecutive (day 1 and 2; day 1 is the day when hyperglycemia was first noted) BGV≥250 mg/dL; for the anti-CD20 plus proinsulin studies the diabetes threshold was >200 mg/dL and hyperglycemia was defined as BGV values >180 mg/dL on two consecutive occasions.

### Anti-CD20 Mab, Oral Insulin, Proinsulin Plasmid, and Anti-CD3 Mab

Anti-CD20 Mab and proinsulin DNA vaccine were obtained from Genentech Inc, (San Francisco, CA) and Bayhill Therapeutics (San Mateo, CA), respectively. Proinsulin plasmid has been described before [9]. The novel anti-CD20 antibody, clone 5D2, is of mIgG2a isotype; the isotype control antibody was IgG2a non-B-cell-depleting C1.18 Mab (Bio-X-Cell). Oral insulin was human recombinant insulin (Humulin R U-500, Eli Lilly & Co, Indianapolis, IN); the diluent given as the oral insulin control consisted of glycerin 16 mg/mL, metacresol 2.5 mg/mL, and zinc oxide (total zinc content 0.017 mg/100 U). Anti-CD3 Mab consisted of the F(ab)2 fragments from the T-cell-depleting anti-CD3 Mab 145-2C11 (hamster IgG).Recent-onset Diabetes Treatments

The anti-CD20 plus oral insulin studies were performed in three independent labs (La Jolla, Denver and Gainesville); all animals (except the anti-CD3 control group) received an insulin pellet implant (LinBit, Linshin Canada, Inc., Toronto, Ontario) on day 1 of confirmed diabetes and a second implant if necessary (2 consecutive BGV≥250 mg/dL). NOD mice with new-onset diabetes were allocated to 6 treatment groups. Group A: isotype control antibody and oral diluent; group B: oral insulin (1 mg twice weekly×6 weeks) starting 2–5 days after diabetes onset; group C: anti-CD20 Mab (10 mg/kg IP on days 1–4); group D: oral insulin plus anti-CD20 Mab (regimens as for groups B and C); group E: same as group D but oral insulin was delayed to start on day 21; and group F: treatment with anti-CD3 Mab (5 µg on days 1–5) as positive control.

For the anti-CD20 plus proinsulin studies, NOD mice were treated with anti-CD20 only, proinsulin plasmid only, or a combination of both anti-CD20 and proinsulin plasmid. In mice treated with anti-CD20 only, varying doses of anti-CD20 (indicated in the figure legends) were given in 200 µL PBS via i.v., tail vein injections, administered four times on consecutive days (days 1–4). In mice treated with proinsulin plasmid only, 50 µg proinsulin plasmid was administered in 200 µL PBS containing 0.9 M CaCL_2_ via i.m. route (100 µL or 25 µg in each large thigh muscle) as described earlier [9]. For combination therapy treated mice, anti-CD20 and proinsulin treatments were given simultaneously; anti-CD20 antibody was given on days 1–4 while proinsulin plasmid was administered once every week beginning on day 1. After treatment with anti-CD20 mono- or combination therapy, BGV measurements were performed until the values were above 250 mg/dL for more than two weeks (no protection) and/or if they stayed <250 mg/dL with no signs of diabetes relapse (protected). Treated NOD mice that showed fluctuations in BGV were monitored for longer time periods to confirm that the mice were either protected or not, sometimes resulting in non-uniform X-axes. In the positive control anti-CD3 treated group, once the diabetes reversal rate stabilized with no mice showing relapse, BGV measurements were stopped, accounting for the difference in follow-up time periods between different treatment groups. In our previous (Bresson D, et.al, JCI 2006) and current experiments (not shown), NOD mice treated with anti-CD3 that have reversed diabetes without relapse for two or more weeks do not relapse, obviating the need for longer-term monitoring.

### Prevention Therapy

Normoglycemic 8–10-week old NOD mice were treated with varying doses (indicated in figure legends) of anti-CD20 alone or in combination with proinsulin plasmid. Anti-CD20 treatments were performed as above in diabetic NOD mice, except that the dose of anti-CD20 used was 10 or 50 µg/mouse in 200 µL PBS. In mice treated with proinsulin plasmid only, 50 µg proinsulin plasmid was administered as above either only once, or four times, or continuously throughout the experiment, at weekly intervals. In combination therapy treated mice, anti-CD20 and proinsulin treatments were given simultaneously; anti-CD20 antibody was given only on day 1 while proinsulin plasmid was administered once every week for four weeks. Unmanipulated NOD mice were used as controls. After treatment with anti-CD20 mono- or combination therapy, BGV measurements were performed until the age of the NOD mice was 40 weeks or until they turn diabetic, to confirm that the protected mice did not turn diabetic with longer observation.

### Flow Cytometry

Flow cytometry was performed as previously described [34]. mAbs specific for anti-IgM, -B220, -CD1d, -CD21, -CD23, -CD3, -CD4, -CD5, -CD8, -CD25, -CD62L, -CD127, IL-4, IL-10, IFN-γ, and control isotypes were purchased from BD Biosciences. Foxp3 staining was performed using Foxp3 staining kit, according to the manufacturer’s instructions (eBioscience, San Diego, CA). Data were acquired on an LSR-II (BD Biosciences, San Diego, CA) and analyzed using Flowjo software (Treestar Inc., NJ).

### Multiplex Cytokine Measurement

Serum was obtained from untreated, anti-CD20 only, or combination therapy treated mice retroorbitally, at weekly intervals. TNF-α, IFN-γ, IL4, IL-10, and IL-17 titers in the serum were determined using a commercially available multiplex cytokine assay kit (Bio-Rad, Hercules, CA) and Bio-Plex 200 instrument.

### B-cell and CD4+ T-cell Purification from NOD Mouse Splenocytes

NOD mouse splenocytes were prepared by homogenization in red blood cell lysis buffer. From pooled splenocytes, B-cells were purified using CD19+ B-cell enrichment kit (Miltenyi Biotec Inc., Auburn, CA). CD4+ cells were purified using CD4^+^CD25^+^ Treg isolation kit (Miltenyi Biotec Inc., Auburn, CA), by stopping the isolation protocol after the depletion step.

### Adoptive Transfers

2–10×10^6^ purified B-cells or CD4+ T-cells from anti-CD20 mono- or combination therapy treated NOD mice were transferred into recipient NOD.SCID mice via tail vein i.v. in 100 µl PBS. The recipient NOD.SCID mice also received an equal number of splenocytes obtained from diabetic NOD mice, in 100 µL PBS (diabetogenic splenocytes).

### ELISpot

ELISpot was performed as previously described [34]. Briefly, purified CD4+ T-cells were prepared from splenocytes and plated in a 96 well PVDF membrane-bound plate (Millipore Inc., USA) coated with cytokine capture antibodies, at 5–25×10^4^ cells/well in triplicate. T-cell-depleted splenocytes (APC) were prepared from 6–8-week old NOD mice and added at 5×10^4^/well along with 50 µg/mL test peptides (proinsulin peptide, and a mutated Insulin B:9–23 B16:A peptide [34]) or medium only. Peptides were used at high purity (>95% by HPLC, Abgent Inc., USA). ELISpot reactions were carried out for 3 days, when the cells were removed followed by the addition of detection antibodies and spot development using 3-amino-9-ethylcarbazole (AEC) substrate (Sigma-Aldrich, USA). Cytokine spots were enumerated using the KS ELISpot reader, software version 4.

### Statistical Analysis

Data are expressed as means±SEM. The statistical significance of the difference between means was determined using the unpaired Student’s t test. Statistical tests were performed using PRISM software (Graphpad, San Diego, CA). For diabetes incidence measurement, statistical significance was determined by Kaplan Meier analysis.

## Results

### Novel Murine Anti-CD20 Preferentially Depletes Follicular and Immature B-cells in the Spleen and Spares Marginal Zone B-cells

Previous studies have shown inconsistent results in replicating the effects of rituximab (anti-human CD20) with murine anti-CD20 antibodies in mice [32]. Here, we investigated the efficacy of a novel anti-CD20 antibody, clone 5D2, in preventing or reversing diabetes in NOD mice, in combination with a proinsulin-expressing plasmid DNA vaccine or oral insulin.

**Figure 1:**
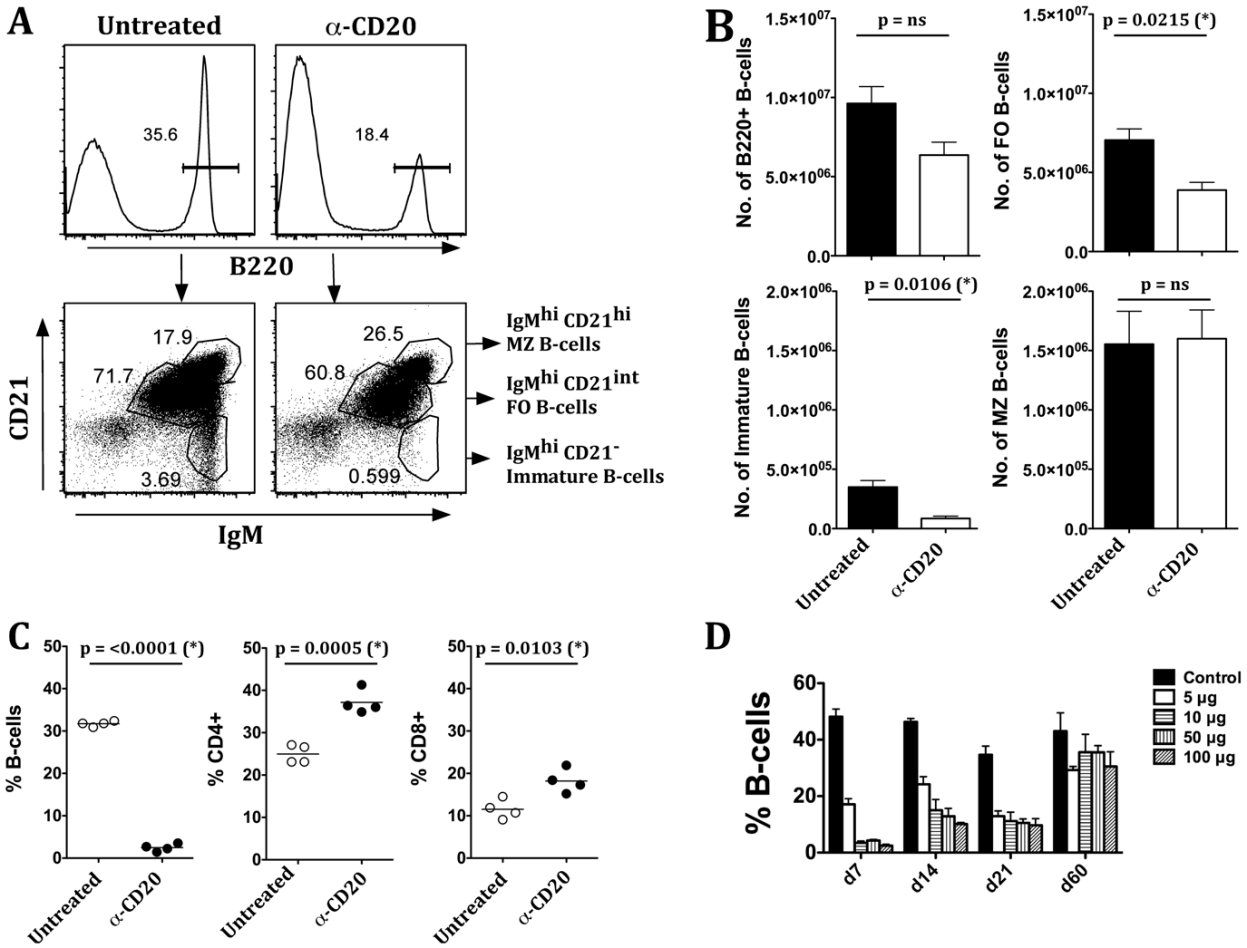
Novel murine anti-CD20 administration preferentially depletes follicular but not marginal zone B-cells. Eight week old female NOD mice were treated with 50 µg anti-CD20 and 7 days later various B-cell subsets in the spleen were analyzed. Gating strategy is shown in the top panel. Lymphocytes were gated on (**A**) B220+ (total B-cells) and further classified as FO B-cells (B220+ IgM^hi^, CD21^int^), immature B-cells (B220+ IgM^hi^ CD21^−^), and MZ B-cells (B220+ IgM^hi^ CD21^hi^). Total B220+ B-cell numbers were reduced in the spleen with significant reductions observed in number of follicular and immature B-cells but not MZ B-cells (**B**). Eight week old female NOD mice were given anti-CD20 i.v. Seven days post anti-CD20 treatment, frequencies of B- and T-cells in peripheral blood were determined by staining with anti-IgM/B220 for B-cells or anti-CD4/CD8 for T-cells; loss of circulating B-cells was associated with compensatory increases in CD4+ and CD8+ T-cell frequencies in NOD mice (**C**). Frequencies of circulating B-cells returned to similar levels as in untreated NOD mice by 60 days post-treatment (**D**). Data are means ± SEM. Representative data from 3 independent experiments with similar results are shown. Statistical analysis was performed using unpaired students *t-*test using Graphpad Prism software.

To determine the efficiency of B-cell depletion using this novel antibody, prediabetic eight to ten week old female NOD mice were treated with a one-time administration of 50-µg/mouse anti-CD20 intravenously (i.v.). Seven days post anti-CD20 treatment, relative proportions and numbers of different B-cell subsets were determined in the spleen by gating on B220 positive B-cells. We observed significant reductions in both frequencies (Fig. 1A) and numbers (Fig. 1B) of total B220+ B cells obtained from spleens of anti-CD20 treated mice, compared to untreated NOD mice. These reductions were found in follicular (FO) B-cells (B220+IgM^hi^CD21^int^) and immature B-cells (B220+IgM^hi^CD21^−^) but not marginal zone (MZ) (B220+IgM^hi^CD21^hi^) B-cells (Fig. 1B). Similarly, efficient depletion of B-cells was also observed in peripheral blood, accompanied by an increase in CD4+ and CD8+ T-cell frequencies (Fig. 1C). The lowest dose tested, 5 µg anti-CD20, achieved 70–80% depletion, while 50–100 µg of anti-CD20 resulted in maximal depletion of B cells (90%) in peripheral blood (Fig. 1D). Following anti-CD20 administration, B-cell numbers were reduced by one week, and stayed reduced for the next two weeks. By eight weeks post treatment, circulating B-cell frequencies returned to levels observed in untreated mice (Fig. 1D).

We attempted to maintain a continuous B-cell depletion in a cohort of NOD mice by giving multiple doses of anti-CD20 antibody separated by either one or three weeks, but these treatment regimens induced rapid death of the mice. Therefore, as perpetual B-cell depletion could not be achieved with the novel anti-CD20 antibody, we administered 50 µg of anti-CD20 on days 1, 2, 3, and 4, which achieved similar levels of B-cell depletion (data not shown). Cytokine analysis of serum samples from anti-CD20-treated animals after one, two, and three weeks post treatment did not show changes in levels of IL-2, TNF-α, IL-4, IL-10 and IL-17 compared to untreated mice (Fig. S1). Taken together, these findings suggest that (a) anti-CD20 administration given either only on day 1 or on days 1, 2, 3 and 4 achieved similar levels of B-cell depletion, and (b) perpetual B-cell depletion could not be achieved in NOD mice using the novel anti-CD20 antibody.

### Transient B-cell Depletion alone or in Combination with Oral Insulin does not Reverse Diabetes in NOD Mice

NOD mice with new-onset diabetes (≥250 mg/dL on 2 consecutive days) were allocated to 6 treatment groups. Group A: isotype control antibody and oral diluent; group B: oral insulin (1 mg twice weekly×6 weeks) starting 2–5 days after diabetes onset; group C: anti-CD20 Mab (10 mg/kg IP on days 1–4); group D: oral insulin plus anti-CD20 Mab (regimens as for groups B and C); group E: same as group D but oral insulin was delayed to start on day 21; and group F: treatment with anti-CD3 Mab (5 µg on days 1–5) as positive control. In all groups, except group F, hyperglycemia was controlled with a subcutaneous insulin pellet on day 1 (repeated if subsequent blood glucose values ≥250 mg/dL on 2 consecutive readings).

**Figure 2:**
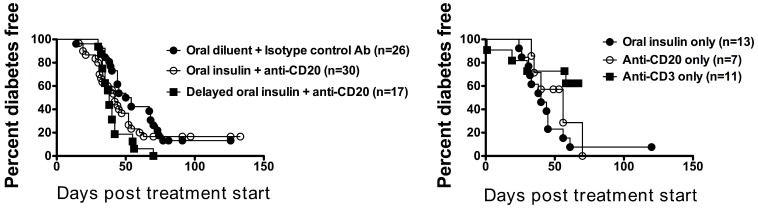
Transient B-cell depletion alone or in combination with oral insulin does not reverse diabetes in NOD mice. NOD mice with recent onset diabetes (2 consecutive readings of BGV>250 mg/dl), were treated with either anti-CD20 alone, oral insulin alone or a combination of both treatments, along with isotype control antibody treatment, oral diluent alone or anti-CD3 mAb treatment, as indicated in figures. These experiments were carried out in three independent labs (indicated in materials and methods) and the combined results were pooled to obtain the results shown here. The number of mice assigned to each treatment group is shown in the figure.

**Figure 3:**
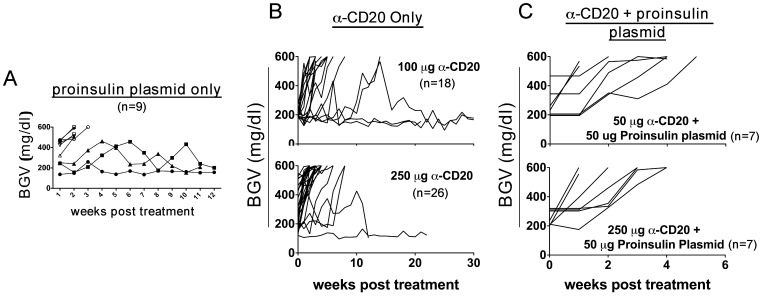
Combination therapy with anti-CD20 and proinsulin plasmid does not offer protection after T1D onset. NOD mice with hyperglycemia (2 consecutive readings of BGV>180 mg/dl), were treated with either (**A**) weekly administration of 50 µg proinsulin plasmid or (**B**) 100 (upper panel) or 250 µg of anti-CD20 alone or (**C**) 50 (upper panel) or 250 µg of anti-CD20 antibody in combination with weekly administration 50 µg of proinsulin plasmid. Each line represents one individual mouse.

Compared to controls (group A), treatment with oral insulin alone (group B), anti-CD20 alone (group C), or the combination (groups D and E) did not induce a significant reversal of hyperglycemia in NOD mice with new-onset diabetes (Fig. 2). In contrast, treatment with anti-CD3 Mab (group F, positive control) resulted in reversal in ∼60% of animals, consistent with published results. To control for the effect of glycemic control with insulin pellets, a separate cohort of mice (see below) was treated with anti-CD20 alone (single injection, either 100 µg or 250 µg per mouse) in the absence of insulin pellets. Again, no consistent reversal of diabetes was noted (Fig. 3B).

Taken together, these results suggest that treatment with a short course of anti-CD20 alone, 6 weeks of oral insulin alone, or the combination, fails to reverse hyperglycemia in new-onset diabetic NOD mice.

### Transient B-cell Depletion Abrogates Proinsulin Plasmid DNA Mediated Protection in Hyperglycemic NOD Mice

To determine the therapeutic efficacy of proinsulin plasmid alone compared to anti-CD20 alone in diabetes reversal, we administered either 50 µg of proinsulin plasmid (pBHT568) at weekly intervals, or anti-CD20 daily for four consecutive days, in a new cohort of hyperglycemic NOD mice (>180 mg/dL; not treated with insulin pellets). Although we did not reach the same level of protection as had been observed previously by Solvason et al., (75%, [9]) 33% (3/9) of mice that received continuous administration of pBHT568 plasmid were protected at 12 weeks post treatment (Fig. 3A). As observed in our first cohort, B-cell depletion with anti-CD20 offered little protection, if any, in hyperglycemic NOD mice (Fig. 3B); only 17% of mice (3/18) that received 100 µg (upper panel) and 8% of mice (2/26) that received 250 µg (lower panel) anti-CD20 showed improvement.

To determine whether combined administration of anti-CD20 and proinsulin plasmid can offer synergistic or additive protection in hyperglycemic NOD mice, we administered anti-CD20 antibody on four consecutive days in combination with proinsulin plasmid given at weekly intervals. Consistent with the oral insulin data, this combination did not offer any protection in hyperglycemic NOD mice (Fig. 3C). Thus, transient B-cell depletion, with or without proinsulin plasmid DNA vaccination, did not offer therapeutic efficacy in hyperglycemic NOD mice.

### Transient B-cell Depletion with Anti-CD20 in Combination with Proinsulin Plasmid Administration Offers Protection from T1D Onset

**Figure 4:**
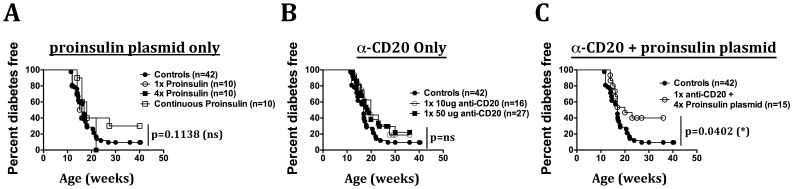
Combination therapy with anti-CD20 and proinsulin plasmid prevents T1D more efficiently. Eight to ten week old prediabetic NOD mice were treated with (**A**) weekly administration of 50 µg proinsulin plasmid only or, (**B**) one-time administration of anti-CD20 alone or, (**C**) anti-CD20 administered in combination with four weekly administrations of proinsulin plasmid. Statistical tests were performed by Kaplan-Meier analysis using Graphpad Prism software.

To test its efficacy in prevention of diabetes onset in NOD mice, proinsulin plasmid was administered in 8–10-week old prediabetic NOD mice once, four times, or continuously, at weekly intervals. Among these different treatment regimens, we found that only continuous administration of proinsulin plasmid showed a trend for protecting NOD mice from diabetes onset (not significant; Fig. 4A). Administration of proinsulin plasmid only once or for four times had no effect on diabetes development in NOD mice.

To determine whether transient depletion of B-cells could protect NOD mice from diabetes onset, multiple cohorts of 8–10-week old NOD mice were given a one-time administration of varying doses of anti-CD20 antibody (10, 50, 100 or 250 µg per mouse) and diabetes progression was monitored. Single administration of anti-CD20 antibody had no significant effect on diabetes onset (Fig. 4B, and data not shown). There was also no significant protection from diabetes onset when anti-CD20 was given four times (on days 1–4, data not shown). Thus, transient B-cell depletion with anti-CD20 does not have any protective effect on diabetes onset in NOD mice.

To determine whether the combination therapy can offer protection from diabetes onset, 8–10-week old prediabetic NOD mice were treated with a low-dose (50 µg) one-time administration of anti-CD20 antibody in combination with four weekly injections of proinsulin plasmid. In contrast to anti-CD20 or proinsulin plasmid alone, combination therapy resulted in a reduced incidence of T1D in NOD mice (Fig. 4C). More frequent administration of anti-CD20 (on days 1, 2, 3 and 4) or administration of a higher dose of anti-CD20 did not increase the level of protection observed (data not shown). Therefore, transient B-cell depletion with low-dose anti-CD20 in combination with proinsulin plasmid offers modest protection from T1D onset in NOD mice.

### Combination Therapy did not Induce Regulatory B-cells

**Figure 5:**
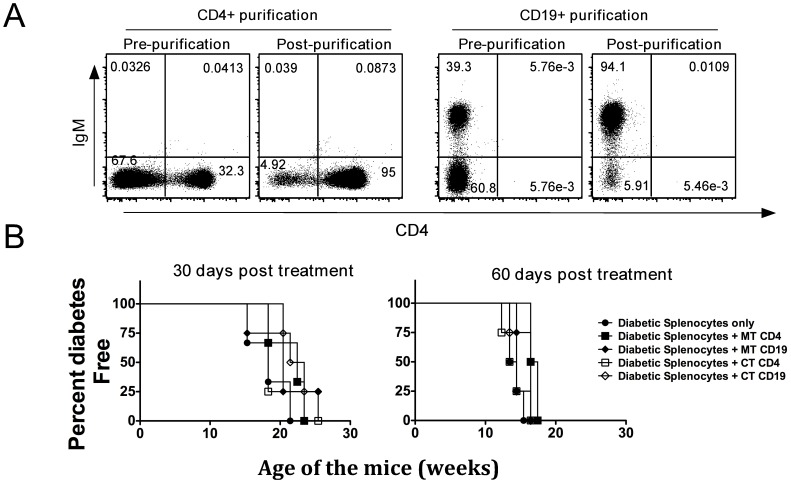
Combination therapy did not induce B-cells with regulatory function. Eight week old NOD mice were treated with anti-CD20 alone (monotherapy, MT) or anti-CD20 in combination with proinsulin plasmid (combination therapy, CT). At 30 days (B; left panel) or 60 days (B; right panel) post treatment (∼16 wks of age) treated non-diabetic NOD mice were sacrificed and B-cells and CD4+ T-cells were purified, from pooled splenocytes (n = 5) by positive selection using magnetic beads. Simultaneously, splenocytes were obtained from diabetic NOD mice. (**A**) Purity of B- and T-cells is shown. (**B**) 2×10^6^ diabetic splenocytes (left panel,) or 10×10^6^ diabetic splenocytes (right panel) were injected into 8-wk old NOD-SCID mice (4 mice per experimental group) either alone, or in combination with 3×10^6^ B- or T-cells purified from anti-CD20 mono- or combination therapy treated mice (as indicated in figure legend). B-cells or CD4+ T-cells from either monotherapy (MT) or combination therapy (CT) mice failed to protect NOD-SCID mice from diabetes onset.

Regulatory B-cells can either be defined by expression of certain cell surface markers (phenotype) or by their ability to suppress T cell activity through IL-10 production or Fas-FasL interaction (function) (discussed in [35]). Using all three phenotypic characterizations [35], we did not detect any increases in regulatory B-cells following anti-CD20 mono- or combination therapy (data not shown). Next, we explored the possibility that functional B-regulatory cells with T cell suppressive activity (not restricted to above phenotypes) were induced upon anti-CD20 treatment. As it is difficult to isolate only functional B-regulatory cells, at four or eight weeks following treatment initiation we purified all B-cells from spleens of anti-CD20-treated non-diabetic mice (with or without proinsulin plasmid co-administration), and adoptively transferred them into NOD.SCID mice along with diabetogenic splenocytes from control NOD mice. B-cells from mono- or combination therapy treated mice did not alter the course of diabetes development in recipient NOD.SCID mice (Fig. 5A, B).

### Combination Therapy Induces Modest Increases in Tregs in Pancreas Draining Lymph Node

**Figure 6:**
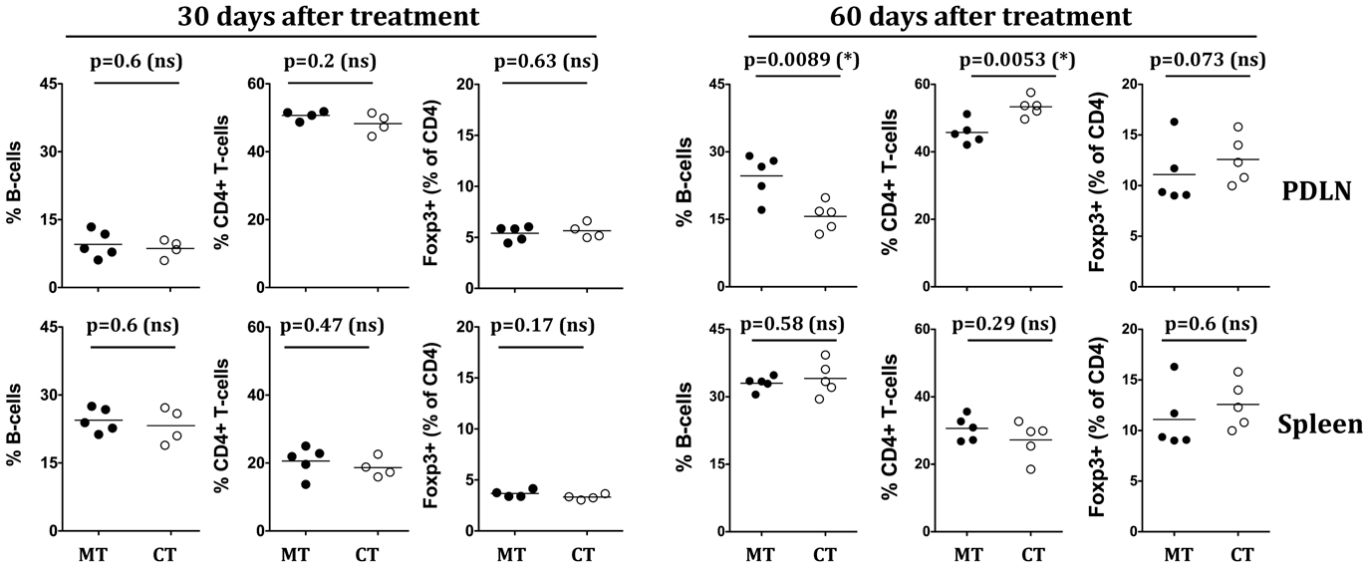
Combination therapy increases numbers of CD4+ Foxp3+ cells in pancreas draining lymph node 60-days post treatment. Eight to ten week old prediabetic NOD mice were given 50 µg of anti-CD20 either alone or in combination with weekly administration of 50 µg proinsulin plasmid for four weeks. Splenocytes and PDLN cells from mono- or combination therapy treated mice were stained with anti-IgM, -B220 (B-cells), -CD4, -CD8, -CD25 and –Foxp3. B-cell frequencies were determined by gating on IgM+ B220+ cells. Frequency of Foxp3+ cells among CD4+ T-cells was determined by gating on CD4+ cells. Cumulative frequencies of B-cells (left panel), CD4+ T-cells (middle panel), and CD4+Foxp3+ cells (right panel) in PDLN (upper panels) or spleen (lower panels) are shown at 30 days and 60 days post combination therapy. Representative data from three independent experiments with similar results is shown. Statistical analysis was performed using unpaired students *t-*test using Graphpad Prism software. Each dot represents one mouse, with mean value indicated on the graph.

We next sought to determine if phenotypic or functional Tregs are induced upon mono- or combination therapy. At eight weeks, but not at four weeks post treatment, there was a modest increase in CD4*+*FoxP3*+* cell frequencies in PDLNs of combination therapy treated mice in comparison to mice that received monotherapy, but this did not achieve statistical significance (Fig. 6). At eight weeks post therapy there were significant decreases in B-cells accompanied by increases in CD4+ T-cells in PDLN but not in spleens of combination therapy treated mice. Since protection offered by combination therapy was sustained long-term (Fig. 4), but there was only a modest increase in Tregs in spleen or PDLN at eight weeks, we sought to determine whether this protection could be mapped to induction of dominant tolerance in all CD4+ T-cells. At four weeks or eight weeks after combination therapy, purified CD4+ T-cells (purity shown in Fig. 5A) from protected mice were adoptively transferred into eight-week-old NOD.SCID mice along with an equal number of splenocytes from diabetic NOD mice. Purified CD4+ T-cells from mono- or combination therapy treated mice failed to protect NOD.SCID from T1D onset (Fig. 5B), suggesting a lack of dominant tolerance induction in CD4+ T-cells.

### Combination Therapy Increases the Number of Proinsulin-specific IL-4-producing CD4+ T-cells

**Figure 7:**
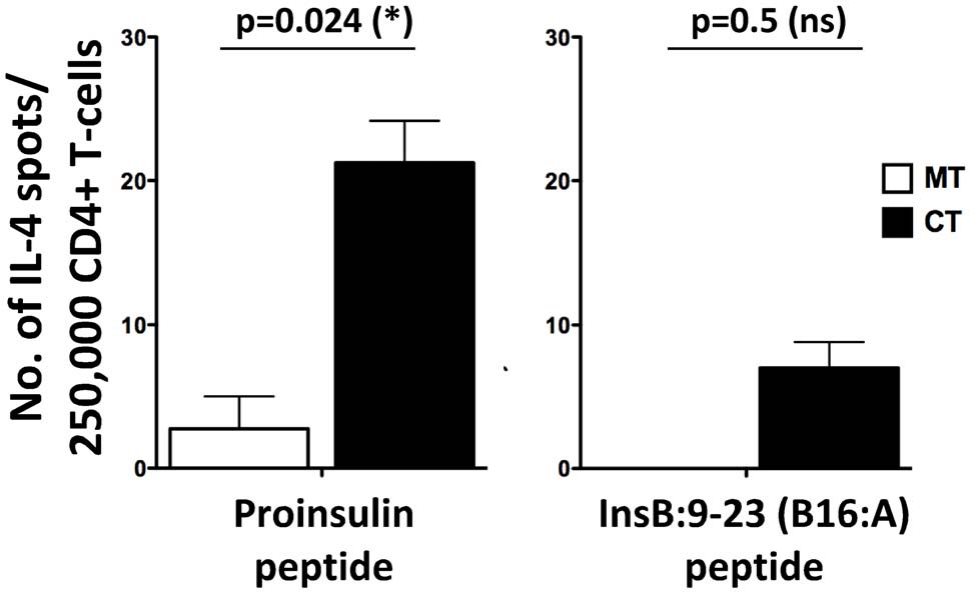
T-cells from combination therapy treated mice produce IL-4 in response to proinsulin peptide stimulation. At 60-days post anti-CD20 mono- (MT, open bars) or combination therapy (CT, filled black bars), CD4+ T-cells were purified from treated non-diabetic NOD mice. Simultaneously, T-depleted splenocytes (TDS) were obtained from 8–10 week old NOD mice and used as APCs. 250,000 purified CD4+ T-cells were incubated with proinsulin peptide or a mutated insulin B:9–23 (B16:A) peptide in the presence of 5×10^4^ APCs. Following 3-day incubation, IL-4 production was determined by ELISpot assay as previously described [34]. Background (media) subtracted spot numbers are shown on the Y-axis. Increased IL-4 production in response to proinsulin peptide was seen in combination therapy treated mice. Representative means ± SEM data from one of two independent experiments with similar results are shown. Statistical analysis was performed using unpaired students *t-*test using Graphpad Prism software.

We have shown that antigen-specific DNA vaccination with plasmid expressing an insulin B chain peptide, InsB:9-23, offers protection via IL-4 production [17]. Therefore, we sought to determine whether proinsulin-specific immune responses were modulated with the combination therapy. At two and eight weeks post anti-CD20 treatment with or without proinsulin plasmid administration, purified CD4+ T-cells were tested for their ability to respond to proinsulin peptide stimulation in an ELISpot assay. Purified CD4+ T-cells were stimulated with either proinsulin peptide or a modified InsB9-23 peptide (B16:A [13]). At two weeks post treatment, CD4+ T-cells from anti-CD20 mono- or combination therapy treated mice did not produce any IL-4 in response to proinsulin peptide stimulation (data not shown), suggesting a lack of proinsulin specific T-cell expansion at this time point. Interestingly, at eight weeks post combination therapy, a significantly higher number of CD4+ T-cells produced IL-4 upon proinsulin peptide stimulation in comparison to mice treated with anti-CD20 alone (Fig. 7). Further, CD4+ T-cells from combination therapy treated mice also produced IL-4 in response to stimulation with a mutated InsB:9–23 (B16:A) peptide (Fig. 7), although the responses were low and did not achieve significance. Taken together, these results suggest that increases in IL-4-producing CD4+ T-cells could potentially contribute to the observed protection with combination therapy.

## Discussion

A recent trial of rituximab in new-onset T1D led to preservation of C-peptide secretion for a period of approximately nine months [21]. Similarly, administration of a proinsulin DNA vaccine has shown promising results in NOD mouse studies [9] and is currently being tested in a phase I/II clinical trial [36]. However, it is evident from over 40 clinical trials conducted in the last three decades that the use of a single immunotherapeutic agent is insufficient to confer protection in a majority of T1D patients and that combination approaches will be required (discussed in [4]). Thus combination therapies with multiple therapeutic agents need to be tested in preclinical studies to accelerate their translation into human trials. In this study, we combined the administration of a B-cell depleting anti-CD20 antibody with either proinsulin plasmid or oral insulin to determine the efficacy of these combinations in protecting hyperglycemic NOD mice. Our results show that neither combination offers protection after onset of hyperglycemia, but that anti-CD20 plus proinsulin plasmid prevents diabetes onset in a modest but significant proportion of animals. These observations provide insights for the design of future clinical trials involving a combination of anti-CD20 and insulin-based antigens.

Previous studies evaluating anti-CD20 antibody to deplete B-cells have found varying effects on T1D onset. While one group found therapeutic efficacy in transgenic NOD mice that express human CD20 [30], other groups failed to observe any protection after T1D onset [32]. A recent study found that continuous depletion of B-cells in NOD mice offered protection from T1D onset in IAA-negative NOD mice [31]. However, because repeated administration of our novel anti-CD20 antibody separated by more than one week led to the death of NOD mice, our own observations are limited to transient depletion of B-cells. Such transient B-cell depletion did not protect NOD mice from T1D onset.

In agreement with Serreze et al. [31], we found that most of the B-cells that entered the pancreas lost their CD20 expression (data not shown) and therefore were not depleted after anti-CD20 treatment. In addition, we found that anti-CD20 treatment did not have any effect on circulating levels of IAA (Fig. S2), consistent with reports that anti-CD20 treatment depletes short-lived plasma B-cells [37], whereas IAA-is produced predominantly by long-lived plasma B-cells. This lack of long-lived plasma B-cell depletion may explain why monotherapy with rituximab shows only limited efficacy in a majority of T1D subjects [18].

In our experiments, continuous proinsulin plasmid administration offered only limited protection in hyperglycemic NOD mice, less than that observed by others previously [9], possibly due to the autoantibody status of the NOD mice. Solvason et al. used only IAA+ mice in their study [9] while we used all NOD mice irrespective of their autoantibody status.

Anti-CD20 treatment was protective in IAA-negative NOD mice [31] while proinsulin plasmid therapy was effective in IAA-positive mice [9]. In our study we combined anti-CD20 and proinsulin plasmid, and therefore we used mice regardless of IAA status. Whether the lack of therapeutic efficacy in hyperglycemic NOD mice could be attributed to differential effects of these agents depending on the IAA autoantibody status cannot be answered in our experimental setup.

While most anti-CD20 treatments require continuous administration [31], [32] to reduce T1D incidence, we observed limited but significant protection with a one-time anti-CD20 administration combined with four doses of proinsulin plasmid. It appears that this combination therapy can induce long-term antigen (proinsulin) specific tolerance in a proportion of mice, likely through IL-4 production. However, involvement of additional mechanisms playing a role in increased protection cannot be ruled out since increases in IL-10 production were found in NOD mice treated only with proinsulin plasmid [9]. Previous work in our lab showed that DNA vaccination with insulin B:9–23 provided protection in T1D through the production of IL-4 from CD4+ T-cells [17], [18]. Thus it is possible that increased antigen-specific IL-4 production could be a common feature of DNA vaccination-mediated protection in T1D.

Islet antigen-specific Tregs operate locally in PDLNs and islets by recognizing their cognate antigens, thus induction of such Tregs is highly desirable through islet antigen specific therapies. However, while monotherapy with islet antigens such as insulin shows promise in animal models, it has little efficacy in human trials [2], possibly because the duration of immunotherapy was too short or because the complexity of human autoimmune diabetes requires addition of an immune modulator to the antigen. Therefore, to improve efficacy in T1D protection, novel combination therapies have to be optimized, ideally in mice first. We have previously shown that combination therapy with anti-CD3 and proinsulin peptide (hpllp) achieved much better protection in NOD mice with recent-onset diabetes than either of these reagents alone [3].

Using combinations of reagents that are in clinical trials as T1D monotherapies (anti-CD20, oral insulin, and proinsulin plasmid) we only found modest protective efficacy in prevention of T1D onset with the combination of anti-CD20 and proinsulin, but no therapeutic efficacy in hyperglycemic NOD mice with either combination. Our results suggest that transient B-cell depletion using anti-CD20 alone or in combination with proinsulin plasmid or oral insulin are not effective T1D therapeutic strategies. These results further emphasize the importance of rigorously testing novel therapeutic approaches in relevant animal models and underscore the need for identifying novel combination therapeutic approaches that can be moved into the clinic.

## Supporting Information

Figure S1 **B-cell depletion does not result in a systemically altered cytokine milieu in peripheral blood.** Eight to ten week old prediabetic NOD mice were given 50 µg of anti-CD20. At one, two or three weeks post treatment serum was obtained and circulating levels of different cytokines (TNF-α, L-2, IL-4, IL-10 and IL-17) were determined by cytokine-multiplex technology. For each cytokine, the samples were analyzed in triplicates and the mean values are plotted as dot plots with each dot representing one individual mouse. The experiment was repeated twice with similar results and data from one representative experiment is shown.
(TIF)

Figure S2 **Anti-CD20 treatment does not diminish circulating levels of IAA auto-antibodies in NOD mice.** Serum was collected from eight week old NOD mice that were either untreated (A) or treated with anti-CD20 antibody (B) before the initiation of anti-CD20 treatment (at 8-weeks of age) and at 1, 2, 3 and 4 weeks post anti-CD20 treatment. Levels of circulating IAA antibody were determined by radioimmunoassay, at Barbara Davis Center, Colorado. Anti-CD20 treatment did not cause a drop in the levels of circulating IAA autoantibodies.
(TIF)
