# Supplementary figures and images for: Transient B-Cell Depletion with Anti-CD20 in Combination with Proinsulin DNA Vaccine or Oral Insulin: Immunologic Effects and Efficacy in NOD Mice
Source: PLoS One. 2013 Feb 6;8(2):e54712. doi: 10.1371/journal.pone.0054712 (PMC3566105; doi:10.1371/journal.pone.0054712)

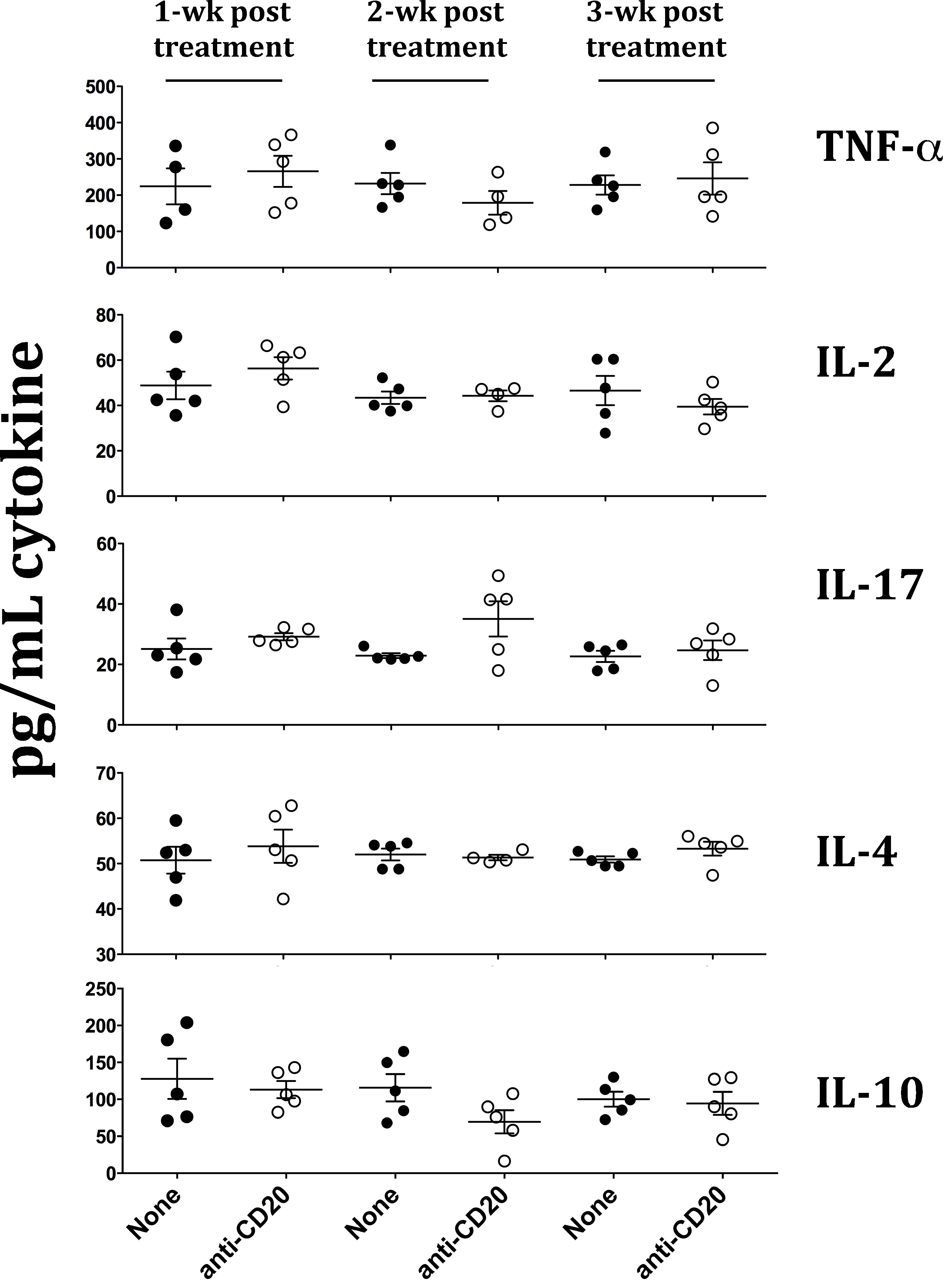

Supplement: Figure S1 — B-cell depletion does not result in a systemically altered cytokine milieu in peripheral blood. Eight to ten week old prediabetic NOD mice were given 50 µg of anti-CD20. At one, two or three weeks post treatment serum was obtained and circulating levels of different cytokines (TNF-α, L-2, IL-4, IL-10 and IL-17) were determined by cytokine-multiplex technology. For each cytokine, the samples were analyzed in triplicates and the mean values are plotted as dot plots with each dot representing one individual mouse. The experiment was repeated twice with similar results and data from one representative experiment is shown. (TIF) [file pone.0054712.s001.tif]

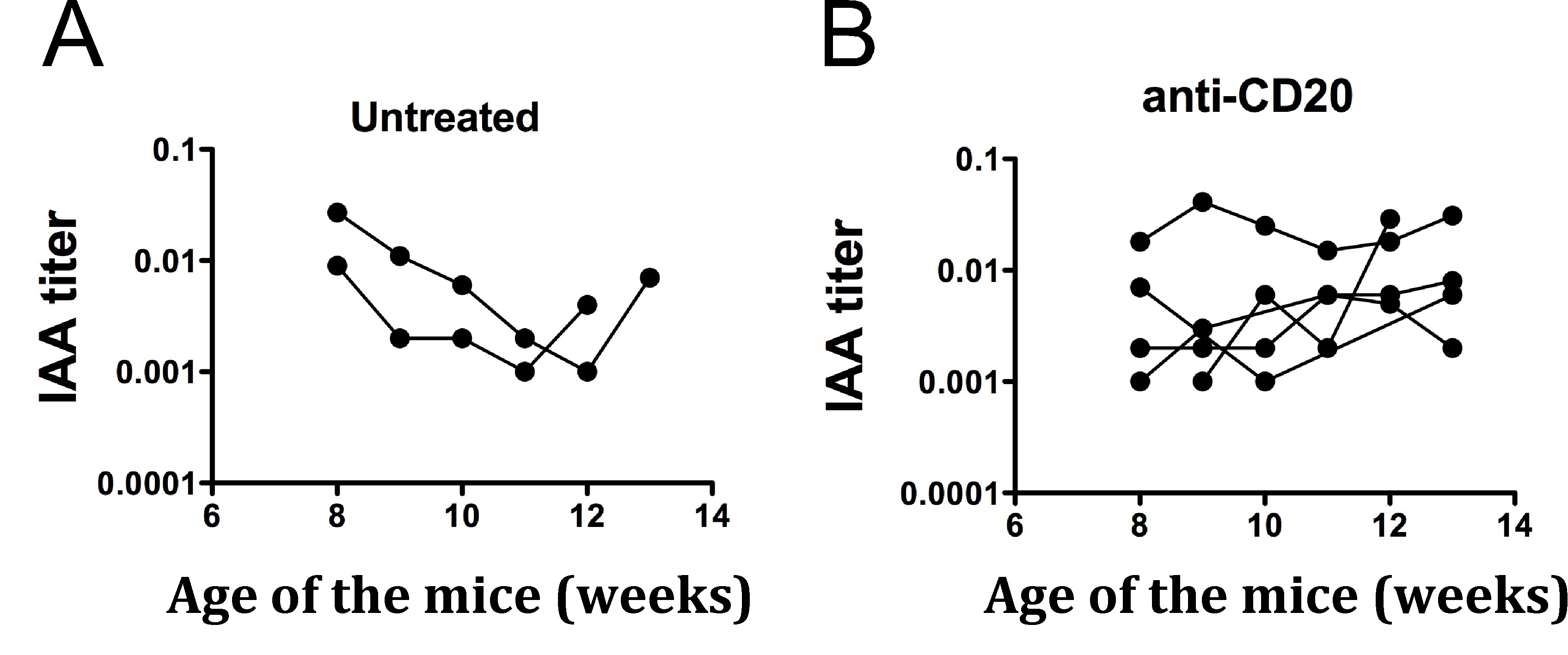

Supplement: Figure S2 — Anti-CD20 treatment does not diminish circulating levels of IAA auto-antibodies in NOD mice. Serum was collected from eight week old NOD mice that were either untreated (A) or treated with anti-CD20 antibody (B) before the initiation of anti-CD20 treatment (at 8-weeks of age) and at 1, 2, 3 and 4 weeks post anti-CD20 treatment. Levels of circulating IAA antibody were determined by radioimmunoassay, at Barbara Davis Center, Colorado. Anti-CD20 treatment did not cause a drop in the levels of circulating IAA autoantibodies. (TIF) [file pone.0054712.s002.tif]
